# Is population structure in the genetic biobank era irrelevant, a challenge, or an opportunity?

**DOI:** 10.1007/s00439-019-02014-8

**Published:** 2019-04-27

**Authors:** Daniel John Lawson, Neil Martin Davies, Simon Haworth, Bilal Ashraf, Laurence Howe, Andrew Crawford, Gibran Hemani, George Davey Smith, Nicholas John Timpson

**Affiliations:** 1 MRC Integrative Epidemiology Unit, Population Health Sciences, Bristol Medical School, University of Bristol, Oakfield House, Oakfield Grove, Bristol, BS8 2BN UK; 2 Institute of Cardiovascular Science, Faculty of Population Health Sciences, University College London, Gower Street, London, WC1E 6BT UK

## Abstract

Replicable genetic association signals have consistently been found through genome-wide association studies in recent years. The recent dramatic expansion of study sizes improves power of estimation of effect sizes, genomic prediction, causal inference, and polygenic selection, but it simultaneously increases susceptibility of these methods to bias due to subtle population structure. Standard methods using genetic principal components to correct for structure might not always be appropriate and we use a simulation study to illustrate when correction might be ineffective for avoiding biases. New methods such as trans-ethnic modeling and chromosome painting allow for a richer understanding of the relationship between traits and population structure. We illustrate the arguments using real examples (stroke and educational attainment) and provide a more nuanced understanding of population structure, which is set to be revisited as a critical aspect of future analyses in genetic epidemiology. We also make simple recommendations for how problems can be avoided in the future. Our results have particular importance for the implementation of GWAS meta-analysis, for prediction of traits, and for causal inference.

## Introduction

### Is population structure relevant in genetic epidemiology?

It could be taken for granted that the problem of population structure (see “Box 1”), in genetic epidemiology, is “solved”. Despite early concerns that phenotypes may be stratified by population (Cardon and Palmer 2003; Freedman et al. 2004; Klein et al. 2005; Marchini et al. 2004) replication rates have been high since the arrival of the genome-wide association study (GWAS) (Pe’er et al. 2008) and consequent adoption of stringent genome-wide significance levels. Phenotype stratification is routinely corrected for using principal components analysis (PCA) (Price et al. 2006) and a range of simple methods (Bouaziz et al. 2011) all appear effective at controlling false positives. State-of-the-art methods use linear mixed models (LMMs, Hoffman 2013; Loh et al. 2015; Zhang et al. 2010) which also control for kinship (“Box 1”). Furthermore, large-scale collaborations in genetic consortia, such as GIANT which examined over 300K individuals in over 100 studies (Locke et al. 2015), enable both replication and the pooling of effect estimates from independent populations. Heterogeneity analyses (Kulminski et al. 2016) are often used to quantify and understand variation.

Indeed, any residual relatedness or familial structure in its broadest sense can now be recruited to help analyses and potentially gain information. The restricted maximum likelihood (REML) method underlying inference in LMMs can be exploited to estimate the “heritability”, or proportion of variation in a phenotype explained by genotyped single nucleotide polymorphisms (SNPs) (Yang et al. 2011). These methods exploit population structure using the genetic relatedness matrix—a particular choice for the measurement of kinship based on SNP similarity—to assess if more genetically similar individuals are more phenotypically similar.

Despite the success of GWAS and heritability analysis, we are entering a new biobank era of massive scale single data collection exercises. Examples of these include 500K participants in the UK Biobank (Sudlow et al. 2015), 500K enrolled into the China Kadoorie Biobank (Chen et al. 2011) and the million veterans program in the US (Gaziano et al. 2016). Importantly, they are of a scale sufficient to both capture signatures of historic demographic events but also be sufficiently influenced by their sampling structure to generate properties in data that can bias association results or their interpretation. This article sets out some reasons to characterize population structure, and specifically:

1. A bias may remain in either direction of the estimated causal effect of a SNP on a trait, after correction for population structure.
2. The effect of correction for structure is a function of the dataset, especially when there is different detection power.

As a consequence:

3. Prediction and heritability analyses require a thoughtful investigation regarding the types of causal pathway that are useful to retain, depending on the intended use of the analysis.
4. Applied analyses such as two-sample Mendelian randomization estimation of the effect of an exposure on an outcome may be biased by population structure when the two samples differ in composition or when they differ in size.

But there are some upsides:

5. Comparison of datasets against a standard reference population structure will resolve many of these issues.
6. Population structure can be very informative about pleiotropy or other biases in causal estimates.

Having argued that population structure is not simply “solved”, this article continues with the following structure. We next address the “challenges” being faced in routine analyses. This begins by defining a goal of correction for population structure and show that it has worked in GWAS, but that there are still open problems in the understanding of selection, Mendelian randomization, and prediction. In “opportunities” we describe ways that population structure can be exploited to learn more about the causal link between genetics and biology, as well as describing methodology that might solve the problems. To validate the high-level claims being made, we will consider simple simulations as well as re-examining examples from the literature. Finally, in the discussion we consider what the problems might imply biologically and give some first steps towards solving them.

## Challenges in population structure and phenotype stratification

In this section, we will demonstrate that in theory and in practice, most methods that use genetic associations are vulnerable to subtle, but important problems that derive from population structure. A key claim in this paper is that *associations* between genetic loci and traits have been reliably established, but *estimates of effect sizes* are less robust. Many uses of population structure depend crucially on unbiased effect size estimates.

The claim that population structure may have been underexplored is not new. It is now understood that structure may have led to different signatures of selection between UK Biobank and the GIANT consortium in height (Berg et al. 2018; Sohail et al. 2018). The problems may not be specific to the study of selection: Berg et al. note that “population structure corrections in GWAS may not always work exactly as expected” whilst Sohail et al. conclude that “polygenic adaptation signals based on large numbers of SNPs below genome-wide significance are extremely sensitive”. Population structure has been recently confirmed as a key part of the problem (Barton et al. 2019; Berg et al. 2019; Sohail et al. 2019), and other authors report residual associations between PCs, geography and traits in the UK Biobank (Haworth et al. 2018; Liu et al. 2018).

### Population structure is correlated with phenotypes

To understand why effect estimates may be biased, it is helpful to revisit ideas in population genetics. Populations do differ genetically by genetic drift and/or selection, and as a consequence these populations will also have different genetic phenotypes. For example, ancient populations had different “genetic heights” (Mathieson et al. 2015), with some potentially being taller than any modern population. Height, and other traits, appear to be “omnigenic” (Boyle et al. 2017); that is, there is no region of the genome not in linkage disequilibrium (LD) with SNPs causal for these traits. Since modern populations are a mixture of older populations, SNPs causal for the trait are themselves correlated with ancestry. It follows that the estimate of the effect of a SNP on a trait can be an underestimate when correcting for population structure.

**Fig. 1:**
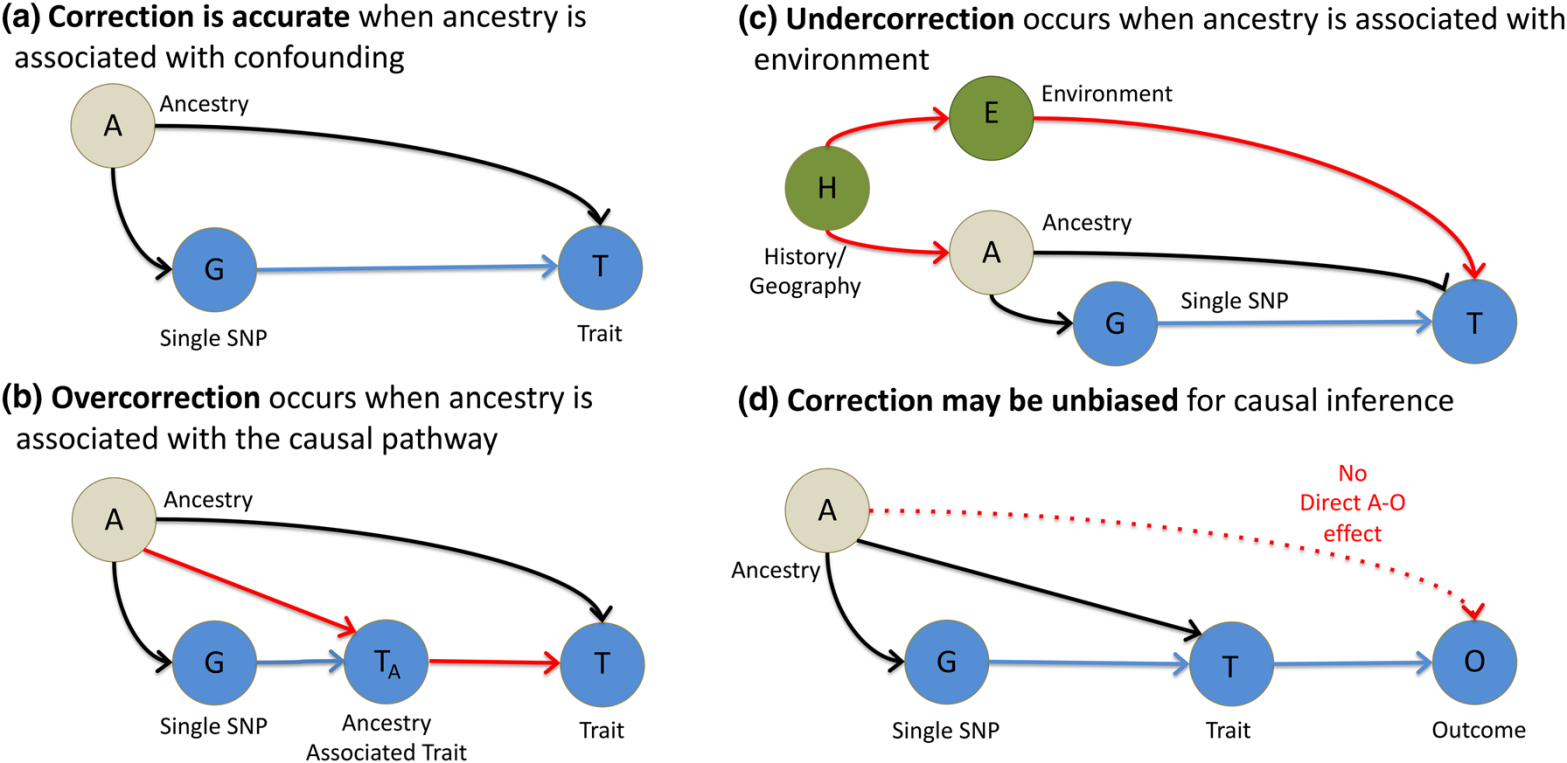
Causal models including ancestry for the effect of a SNP (*G*) on a trait (*T*). **a** Correction for structure will be accurate when ancestry (*A*) is confounding *T*. **b** Correction for structure may give biased inference when ancestry is associated with the causal pathway (*T*_A_, which may not be measured) by which the SNP acts. For example, *T* = skin cancer is associated with *T*_A_ = skin tone. **c** Correction for structure will be incomplete when ancestry is associated with the environment (*E*) due to shared history and geography (*H*), for example *T* = BMI with *E* = diet choice. **d** Correction for structure when using causal inference is robust to complexity, provided the assumptions of Mendelian randomization (see text) are met; particularly all remaining effects of ancestry go through the trait (*T*) so there is no direct effect of ancestry (*A*) on the outcome (*O*)

The justification for PCA correction for population structure (Price et al. 2006) is to correct for *non*-*causal linear associations* between ancestry and phenotype (Fig. 1a). Causality is hard to define because we rarely measure the exact cause, but proxy it; here we are interested in proxies that are genetic and act through biological pathways. A non-causal association can be generated when population structure is associated with both allele frequency and the phenotype (Fig. 1a). For example, genetic drift simultaneously changes phenotype and SNP frequencies by chance. Weak genetic drift as experienced by larger populations over short timescales is additive, which corresponds to an additive effect on PCs (McVean 2009). Larger genetic drift, as produced by extreme bottlenecks or consanguinity, is not additive as the SNP frequency distribution becomes skewed and SNPs may become fixed or lost from a population. PCA correction and related methods are less useful when such drifted populations are included (Lawson et al. 2018).

Admixture can change SNP frequencies genome-wide, and small admixture variation is ubiquitous. Even large modern human populations not homogeneous—each individual has a slightly different ancestry proportion from earlier populations. The most ancient detectible human admixture event—Neanderthal introgression into Eurasians—has a mean of around 2% (Sankararaman et al. 2014), but varies substantially between populations and individuals (Wall et al. 2013). Many features of Neanderthal ancestry can be correctly understood using GWAS, which is associated causally with some phenotypes including increasing the risk of depression (Simonti et al. 2016), and non-causally with others, for example skin color (because Neanderthal genes entered the modern human gene pool outside of Africa).

Admixture has the potential to interact with family studies. Siblings have the same expected value of ancestry, with them both receiving a random realized amount. Realized, rather than expected, ancestry is a better predictor of phenotypes (Speed and Balding 2015). Such admixture variation can tag an environmental covariate, for example alcohol consumption influenced by ALDH2 (Price et al. 2002). It could also tag another phenotype that has a confounding relationship, for example, when mixed-race siblings vary in skin tone they may experience different societal pressures (Song 2010), which would be plausibly associated with educational attainment (Light and Strayer 2002) and other phenotypes. A causal analysis would include this pathway—i.e., in the examples, ALDH2 is causal for alcohol consumption and skin tone for education. However, in the second example the inference does not fit our definition of being biologically caused since it is mediated solely through modifiable societal norms.

Whilst genetic drift can create phenotypic variation between populations, selection does so much more rapidly (Nielsen 2005). If a phenotype is under selection in a particular population, all SNPs that causally affect that phenotype (and also those in linkage disequilibrium) will change in frequency, inducing an association between ancestry and phenotype. Further, where some of the variants affecting a selected phenotype are pleiotropic or in LD with SNPs for another phenotype, selection can generate genetic associations between the phenotype under selection and other phenotypes. In extreme cases selection can lead to allele frequencies being almost perfectly correlated with population structure. The *LCT* gene (Bersaglieri et al. 2004) which is associated with lactase persistence, and similarly a variant in *ADH1B* (Galinsky et al. 2016) which influences alcohol metabolism, both stratify by population.

### Impact on Mendelian randomization

Population structure bias has also been discussed in relation to Mendelian randomization (Davey Smith and Ebrahim 2003; Davey Smith and Hemani 2014; Didelez and Sheehan 2007; Lawlor et al. 2008), an approach which uses a SNP or groups of SNPs as an instrument or “proxy” to test whether an exposure causes an outcome. Mendelian randomization estimates the causal effect under the assumptions (Davies et al. 2018) that; (a) SNPs are associated with the exposure; (b) SNPs do not influence the outcome through a pathway independent of the exposure; (c) that there are no confounders of the SNPs–outcome relationship. Population substructure differences can in theory affect both the strength of genetic instruments and induce confounding, for example in the study of lactase persistence (Campbell et al. 2005; Davey Smith et al. 2009), but there is little evidence the problem is widespread.

The loci that are particularly useful for Mendelian randomization may be particularly susceptible to bias from population structure. This is because strong associations are generated through strong selection, which as discussed above is typically structured. For example, Mendelian randomization studies for alcohol consumption in Europeans typically use the variant in *ADH1B* as a genetic proxy (Holmes et al. 2014; Howe et al. 2019; Lawlor et al. 2013, 2014; Zuccolo et al. 2013). The *ADH1B* variant is associated with ancestry at the country and continental level (Li et al. 2011).

### Understanding ancestry correction

For detection in GWAS, a sensible aim is to have the most stringent control of any potential bias, including for phenotype stratification. In addition to PCA correction for stratification, GWAS has also been controlled using genomic control (Devlin et al. 2001) which accounts for confounding by scaling test statistics using an inflation factor to ensure that “null” SNPs (as represented by the median) behave as expected under the null model. However, if all SNPs have a true effect this approach is under-powered. Linkage disequilibrium can be exploited to separate real from confounding signals, implemented in the popular tools LDAK (Speed et al. 2012; Speed and Balding 2019) and LDSC (Bulik-Sullivan et al. 2015). The premise is that if every SNP has an effect then SNPs that are in regions of higher LD will have larger measured associations because they are composites of their own effects and those around it. These methods confirmed that large-scale GWAS results detect real associations, but what about the size of the effect?

A central goal of genetic association studies is to estimate the “true” causal effect of a SNP (*G*) on a trait (*T*). The “true” effect is defined as the effect of *G* on *T* when all other traits *that are not in the pathway between G and T* (i.e., confounders) are accounted for (Fig. 1). Correction of GWAS for ancestry (*A*) is designed to remove *non*-*causal* associations when observable ancestry (*C*_A_, which might be PCs) not in the causal pathway (Fig. 1a). However, it also removes *causal* associations when ancestry is associated with traits in the pathway (Fig. 1b); a phenomenon often called vertical pleiotropy. Corrected estimates of the *G*–*T* associations exclude the *G*–*A*–*T* association. However, they also exclude the *G*–*T*_A_–*T* association and hence may under-estimate the effect size. For example, if *G* increases the risk of skin cancer by changing skin tone, its effect size will be underestimated if skin tone is predicted by ancestry. In general, because modern populations are mixtures of ancient populations, many SNPs with a biological effect (including ADH1B and Lactase) may associate with ancestry PCs due to having been common in only one ancestral population.

Ancestry can also associate with the environment (*E*) and hence also environmental confounders (*C*_E_) (Fig. 1c). There is no causal relationship between *A* and *T* via *C*_E_ and so *if G is associated with E then correction is desirable* to obtain a less biased estimate of the causal effect of *G* on *T*. However, the measured ancestry *A* is unlikely to account for all association between *E* and *T*, so observing an environmental effect indicates the need for additional phenotyping of that environment. For example, if a culture has a diet that reduces BMI then controlling for ancestry only partially corrects for diet. The same problem occurs if observable ancestry (e.g., PCs) do not completely capture the true ancestry.

### Genome-wide genetic measures are strongly affected by population structure

A SNP–trait association estimate may be biased after ancestry correction when there is a correlation between the (true) SNP–trait effect and the contribution to an ancestry observable from the SNP (e.g., PC loading). There are many SNPs contributing to ancestry measures, so the bias for each SNP–trait estimate is likely to be small, but genome-wide estimates sum this bias. For example, heritability estimates can in theory be biased by population structure through the prediction of non-genetic covariates (Browning and Browning 2011; Dandine-Roulland et al. 2016), though the scale of the problem is not well quantified for most phenotypes. The robustness of heritability estimates to the existence of internal population structure can at least be tested (Speed et al. 2012, 2014).

**Fig. 2:**
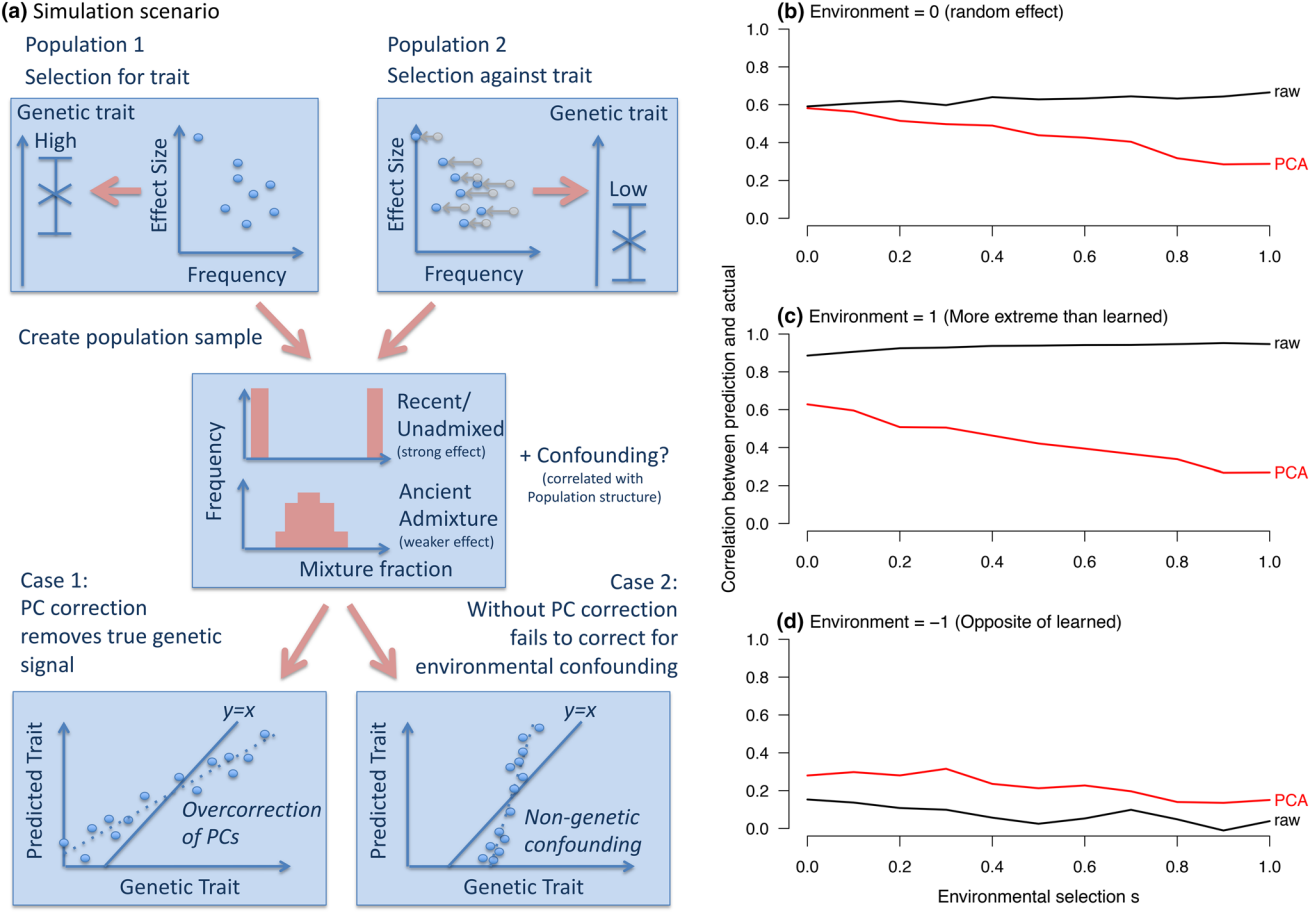
When should we use PCA correction? **a** In simulation settings (see “Methods”) it is straightforward to construct scenarios where correction helps or hinders prediction of traits. Top: two populations are produced with different genetic phenotype, either by drift or selection. Middle: these are mixed to make modern populations. Bottom: in Case 1 the phenotype is associated with true population structure, which can be overcorrected. In Case 2 confounding non-genetic association is included in the prediction. **b**–**d** Show results for this simulation. **b** Correcting for confounding using PCA reduces prediction accuracy when traits are genetically associated with population structure. **c** Genetic structure can predict non-genetic confounding leading to apparently good performance on similarly biased populations. **d** PC correction can protect against this confounding at the cost of reduced performance

Another genome-wide task is to use genetic data to directly predict phenotypes (called genomic prediction, Meuwissen et al. 2001). A predictor is learned using one dataset, then applied to genetic data from others which may be more or less similar in terms of the populations than make it up. This “out of sample” use case makes prediction particularly vulnerable to bias. As demonstrated in Fig. 2, conservative estimates of effect sizes are less useful than a bias–variance tradeoff accounting for the intended populations to be predicted. Adjustment for the PCs is likely to create a higher mean-square error, and it systematically reduces the variance explained in a heritability analysis. The model correcting for ancestry would be preferred for prediction only if (a) it contained enough predictive power to capture real phenotypic differences, and (b) the use case involved generalization into populations for which ancestry may have different effects; for example, predicting skin cancer would be concerning if the predicted population’s skin tone fell outside the range of study population or was caused by different underlying SNPs.

Genetic “prediction … is generally not robust to minor changes in the population” (Goddard et al. 2016). LD in Africans is lower than in Europeans, which makes prediction harder (de los Campos et al. 2010, 2015). A recent study claims that “effect sizes for European ancestry-derived polygenic scores are only 36% as large in African ancestry samples” (Duncan et al. 2018). Yet in consumer genomics (Multhaup and Lehman 2017) and many applications in medicine (Bloss et al. 2011) including drug response (Roden and George 2002), prediction is the primary goal, and ancestry is known to be important (Foll et al. 2014). Prediction is also important for ancient genomics, for example the recent reconstruction of the facial features and dark skin tone of “Cheddar Man” in Neolithic Britain (Brace et al. 2018). Prediction protocols typically involve summing the effect of all SNPs that are reliably associated with phenotype to make a “genetic score”—two early examples include coronary heart disease (Ripatti et al. 2010) and gout (Dehghan et al. 2008). However, other prediction models may be necessary when the genetic architecture of the trait does not follow the infinitesimal and additive assumptions (Morgante et al. 2018).

With the availability of much larger datasets, there is increasing discussion about whether polygenic risk prediction should be included in clinical care. For example, Khera et al. (2018) created a polygenic risk score consisting of millions of variants. The top 8% of the population by this score had comparable risk of coronary heart disease to carriers of rare monogenic mutations. While key coronary heart disease loci such as 9P21 have been replicated worldwide (Battram et al. 2018; Dong et al. 2013; Kral et al. 2011; Schunkert et al. 2011), the generalizability of polygenic risk scores of millions of SNPs across different populations requires further study.

### Interaction between Mendelian randomization and population structure

Causal inference via Mendelian randomization exploits the effect of *G* on *O* that goes via the trait *T*. If the assumptions are met, Mendelian randomization estimates are robust to bias in *G*–*T* estimates, as long as there is no (uncorrected) direct *A*–*O* effect. One important mechanism by which *G*–*O* (gene–outcome) associations might go via A is linkage disequilibrium (LD). If the instrument SNP G is only a proxy for the true causal SNP, and LD differs between populations, then in theory G can be a strong genetic instrument in one population but weak in another. There is an absence of evidence for this phenomenon, likely due to a lack of large African datasets for whom LD is very different than Europeans.

In recent years, Mendelian randomization studies have increasingly used a two-sample design (Hartwig et al. 2016); in which estimates of the SNP–exposure and SNP–outcome relationships are taken from separate GWAS using non-overlapping samples. Here, an implicit assumption is that the two different samples used to estimate these relationships are drawn from the same underlying population. Typically, the two-sample design will use individuals of similar ancestry (e.g., restricting to individuals of recent European ancestry), however the effect of using two similar but ancestrally distinct samples within a broad definition such as Europeans is currently unclear. We will show below that even if the two samples are from the same population, there may be less power to detect the PCs in a smaller population. This can theoretically result in differential correction and, as a consequence, may bias causal estimates.

### Simulating phenotypes with population stratification

For genome-wide questions including heritability analyses and prediction, it is easy to construct scenarios in which either correction or non-correction for structure can be misleading. Figure 2 describes two simulation scenarios: case 1 in which true genetic signal for a trait is associated with population structure (e.g., height), and case 2 in which population structure associates non-causally with the trait through the environment. PC correction is conservative when phenotypes are truly associated with ancestry (Fig. 2b). When ancestry is predictive of the environment (Fig. 2c) it can even increase genetic associations through non-causal pathways. However, when genes have moved into new environments, PC correction reduces bias (Fig. 2d).

### Detecting population structure is essential for correcting for it

If not appropriately modeled, phenotype stratification can bias GWAS, heritability estimation, prediction, and Mendelian randomization. However, no single bias-correction approach is necessarily the correct choice for all scenarios. Even if the correct strategy is known, measurement of population structure is critical. As with any parameters estimated from a dataset, increasing sample size increases the ability to detect population structure (Patterson et al. 2006). Within the UK, there was no detectible structure in a subset of around 1000 people from the UK10K project (The UK10K Consortium 2015). However, with over 100,000 people (Galinsky et al. 2016) from the UK Biobank project (Sudlow et al. 2015) several axes of variation are visible in the PCs. Importantly, the latent structure proxied by these axes of variation were still in the data before they could be detected, and so correction on smaller datasets will systematically under-correct for stratification. This may explain why estimates from a single large study are different from a meta-analysis of smaller ones, though to our knowledge this has not been studied.

To the extent that detection of population structure is a problem, better methodology offers a solution. Methods based on “chromosome painting” (Lawson et al. 2012) exploit linkage disequilibrium to better detect population structure. Specifically, the approach counts recent sharing of segments of DNA that are identical by descent, rather than SNP frequencies, to detect recent structure. From the 2039 individuals in the People of the British Isles (PoBI) dataset (Leslie et al. 2015) there was only 1 geographically meaningful PC but over 50 populations detectable with chromosome painting. Studies sampled from a single location such as many cohort studies (for example ALSPAC, Boyd et al. 2013) are typically PCA corrected but the PCs are too weak to capture the real variation and thus the population is assumed to be “homogeneous”. There is a “detection threshold” in ancestry for which we can calculate the sample size required to detect ancestry variation of a given size (McVean 2009). To our knowledge, there has been no systematic study of the importance of residual population structure in small samples.

**Fig. 3:**
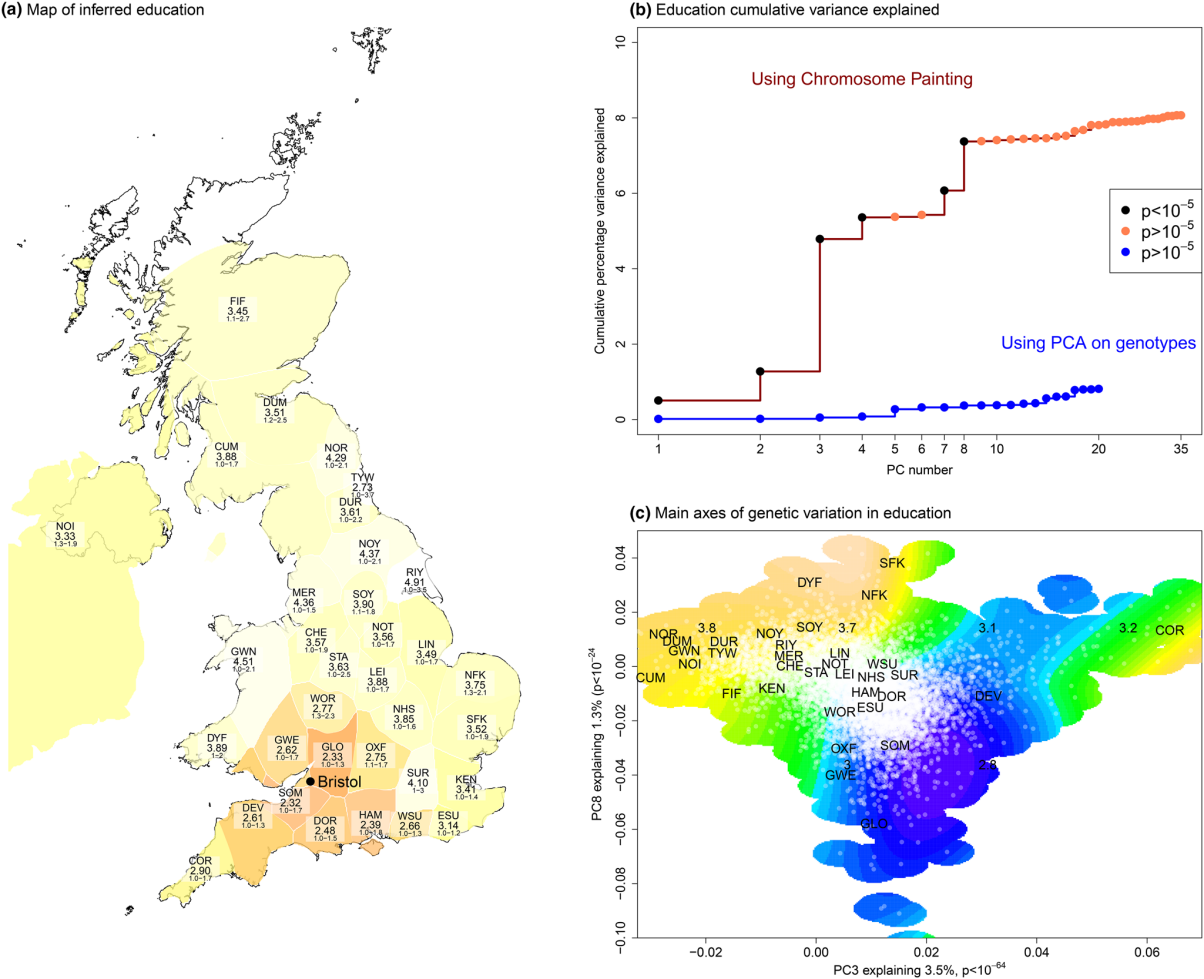
Population structure can be detected in ALSPAC using the external UK reference dataset PoBI and chromosome painting (see “Methods”). This structure is associated with phenotype, and is not found using regular PCA. **a** Inferred (see “Methods”) education level of people migrating from different regions of the UK into the ALSPAC cohort based in Bristol; scale is 1 = no education, 2 = vocational, 3 = GSCEs (age 16), 4 = further education (age 18), 5 = degree (reproduced from Haworth et al. 2018). Participants with ancestry further from Bristol have considerably higher education, suggesting differential migration by education. **b** Variance explained in education by chromosome painting PCs (8%) and regular PCA (0.8%). **c** The chromosome painting PC locations of individuals and populations for chromosome painting PC 3 and 5, which have the largest associations with education. PoBI mean label locations are shown, along with ALSPAC individuals (white dots) and a kernel smoothing of education

Exploiting the PoBI dataset, chromosome painting in ALSPAC (Haworth et al. 2018) (Fig. 3) reveals dramatic genetic heterogeneity which is associated with phenotype, here shown for educational attainment. In this case, the bias is predominantly associated with migration: people who move are more likely to be educated. In ALSPAC, genetic ancestry can predict 8% of the variation in education; for comparison, the most recent published whole-genome genetic score explains 3.2% (Okbay et al. 2016), and a mega-scale analysis is expected to generate a genetic score explaining 10% of the variance (Martin 2018). These results are based on meta-analyses of many studies, in which PC correction may not have sufficiently controlled for population structure.

It is unclear how many of these GWAS hits are in fact hits for ancestry and hidden population structure or migration. The problem exists in many other phenotypes (Martin et al. 2017): for example “height is predicted to decrease with genetic distance from Europeans” which is not empirically observed. Interpretation of these results is left to the discussion.

## Opportunities from structured populations

### Opportunities from natural genetic drift experiments

There are many inferential opportunities offered by population structure, of which two are widely exploited. The first is natural experiments caused by genetic drift. Phenotypic variation decreases on average with distance from Africa (Manica et al. 2007), but any given phenotype may experience extreme variation in a small population. A classic example is the Kosrae islanders in the middle of the Pacific (Lowe et al. 2009) who are at high risk of type 2 diabetes. Similarly, Greek islanders vary dramatically in longevity (Panoutsopoulou et al. 2014) and Ashkenazi Jews (Levy-Lahad et al. 1997) are at high risk of breast cancer; in all three cases, examining drifted populations has led to better understanding of disease for a wider population. In a more extreme example, only 85 individuals were required to identify the gene responsible for blonde hair in Melanesians (Kenny et al. 2012).

**Fig. 4:**
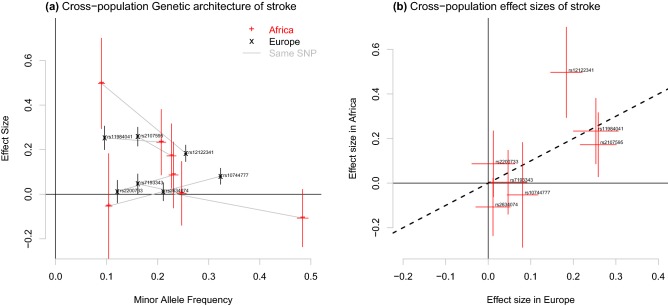
Genetic architecture of significant stroke SNPs, from the GWAS meta-analysis of data from Pulit et al. (2016). **a** Compares minor allele frequency against inferred effect size for Africans and Europeans (larger sample size). **b** Compares the effect sizes only. Effect SNPs are chosen to ensure that the effect directions in the meta-analysis are positive

Exploiting genetic drift in genetic epidemiology is not limited to extreme founder events such as Finland (Cannon et al. 1998) but is actually routinely (if incidentally) used. The well-studied European populations experienced the out-of-Africa bottleneck as well as further founder events (Pagani et al. 2016) and are (due to availability) oversampled. Whilst much variation is missing in Europe (1000 Genomes Consortium 2015), the benefit is that some variants are at higher frequency than selection would allow in the larger effective population size within Africa. Figure 4a describes the GWAS results of a meta-analysis of stroke, for individuals with European, African and Asian ancestry sampled worldwide (Pulit et al. 2016). Seven SNPs were significant in the meta-analysis, which we consider in the context of European or African ancestry. Genetic drift has changed the genomic architecture of the disease; two significant SNPs have increased massively in frequency in the (more drifted) Europeans. Under the assumption that this is drift and not European-specific selection, there may not have been power to detect them in the same sized sample of Africans.

### Opportunities from replication in varied populations

The second widely exploited opportunity from population structure is replication. Replication—or more generally, joint analyses of independent datasets (McCarthy et al. 2008)—is justifiably required for a GWAS result to be accepted. However, replication should be seen as a function of the properties of the populations being studied; Li and Keating (2014) list 11 examples where trans-ethnic replication has contributed to better understanding of GWAS results. When either the environment (Kaufman et al. 2013; Zhang et al. 2015; Logue et al. 2011) or the genetics (as in European meta-analyses) do not differ substantially then confounding can still lead to an observed association by the same mechanism as that which produced the association in the discovery dataset. Examining these in detail can prove insightful (Zhang et al. 2015). In the stroke example, Fig. 4a shows that 3 of the SNPs replicate in Africans, despite lower power in that population, which increases confidence in their association. Less reassuring are the 2 SNPs with different direction of effect, however, these results are imprecise and these differences could be due to estimation error. The current focus on European datasets appears to primarily probe variation and mutations from after the out-of-Africa bottleneck (Simons et al. 2018), meaning that it misses much biology from the critical period of the evolution of humans, as well as having negative implications for genetic applications in medical science (Oh et al. 2015; Martin et al. 2018).

Population structure can be exploited to reach additional biological insights. The true effect sizes may differ between populations, either due to gene–gene or gene–environment interactions. For example, decanalization (Gibson 2009) is a relatively common form of genetic interaction involving the removal of mechanisms that regulate the genome, allowing larger effects. Correct estimation or overcorrection (Fig. 1a, b) are both possible scenarios when genetic variation is associated with ancestry via a gene–gene interaction. Under-correction (Fig. 1c) may occur when genetics is associated with the environment via a gene–environment interaction. However, the scenario can be detected and hence properly modeled by observing genotypes in multiple genetic and physical environments; see for example Vrieze et al. (2012) who examine gene–environment interactions in psychiatric disorders.

In the stroke example in Fig. 4b, 1 SNP (rs12122341) has different size of effect in Europeans and Africans. This is interesting and important as it must be associated with a difference in the genetic or environmental background of the two populations. This means the SNP will violate the Mendelian randomization assumptions within one or both populations, but examining multiple populations allows this to be detected. Hypothetically, the difference in effect seen in stroke may contribute via some other phenotype such as smoking behavior or diet.

### Opportunities from population-aware methodology

So far, we have considered what current methodology and/or simple data analysis can show. Comparisons are therefore SNP-wise, focused on strongly associated SNPs, and usually result in falsifying hypotheses formed from single population analyses. Completely new information can be extracted by instead comparing sets of SNPs or whole genetic architectures (Timpson et al. 2018) across population structures and traits (Simons et al. 2018).

Variation between populations can be exploited as part of the statistical methodology, to further learn about the genetic structure of a phenotype. Within a population, admixture mapping was an early tool (Winkler et al. 2010) to exploit variation in ancestry, though there are relatively few recent novel discoveries using this method, one being Adhikari et al. (2016). Across populations, standard GWAS methodology has been successfully applied and extended in “trans-ethnic” approaches (Li and Keating 2014) which start by treating ancestry as a fixed or random effect in regression. More sophisticated approaches such as MANTRA (Morris 2011) and MR-MEGA (Mägi et al. 2017) model heterogeneity in ancestry-specific effects, allowing the agreement between different populations to be measured. Popcorn (Brown et al. 2016) allows this to be done using only GWAS summary statistics. The consistent story across all phenotypes studied in these papers—rheumatoid arthritis, type 2 diabetes, gene expression, and kidney function—is that that both environment and ancestry play an important a role in explaining differences in populations. The total contribution of both is usually on the same order of magnitude. Although not conclusive in human studies, gene–environment interactions have also been explicitly measured and can contribute substantially, e.g., adding 11% to accuracy in a plant study (Desta and Ortiz 2014).

Current methods for trans-ethnic analysis perform the association and heritability stage, but they can also be used for causal inference. The Mendelian randomization framework can be extended to consider the graph of how all traits may be causally related to all other traits (Hemani et al. 2017). We have seen that access to estimates from multiple populations provides insights into the effect sizes of individual SNPs for individual traits. New methodology should be able to exploit differences across populations to automatically screen SNPs and create causal graphs unique to each population.

## Discussion

### Population structure is relevant for epidemiology

Population structure has always been a feature of genetic studies of phenotypic variation. The impact it has had on inference has varied considerably as the data and questions have changed. Structure confounded the early efforts of genetic discovery, but was then sidestepped by larger datasets and a focus on discovery and replication. Population structure is transitioning from a theoretical problem to a practical issue for questions that require an accurate estimate of effect sizes. This is especially important for prediction where out-of-sample target populations must be considered and Mendelian randomization for which sample size provides a potential source of bias.

### Population structure is still a challenge

The very nature of population structure is challenging, requiring approaches that are specific to the analytical context and trait. Figure 2 demonstrates that the use of stratification correction in larger datasets may overcorrect, whilst Fig. 3 implies that meta-analysis of smaller datasets will systematically under-correct for population structure. The development of new tools to address and exploit structure is an important challenge for epidemiology.

The educational attainment results highlighted by structure within the ALSPAC study reveal important complexity. The effect sizes in trio studies (Okbay et al. 2016) are theoretically not confounded by population structure, and are consistently 30–40% smaller than the inferred effects for the larger, unrelated sample. Our results show that population structure alone can predict educational outcome better than was previously thought. It is still unclear how this predictive power arises—this study implicates migration whilst other explanations include assortative mating and dynastic effects (Kong et al. 2018; Young et al. 2018), as well as sampling biases, though these are not mutually exclusive. We have discussed reasons to adjust GWAS results—or not—using higher quality ancestry estimates for the consortium datasets. There are two opposing hypotheses, which are both consistent with the available data:

- Educational attainment is associated with ancestry because of causal pathways that should be included in our definition of the phenotype. For example, historical biased migration could create “brain drain”, or selection on ancestral populations leading to a difference in ability (Clark and Cummins 2018). Alternatively, phenotypic differences between populations might exert influences over life-choices.
- Educational attainment is associated with ancestry because of non-causal phenotypic pathways. Examples include access to education, cultural norms, the relationship between education and GDP (Nelson and Phelps 1966), and discrimination within the educational system (Light and Strayer 2002; Song 2010).

**Fig. 5:**
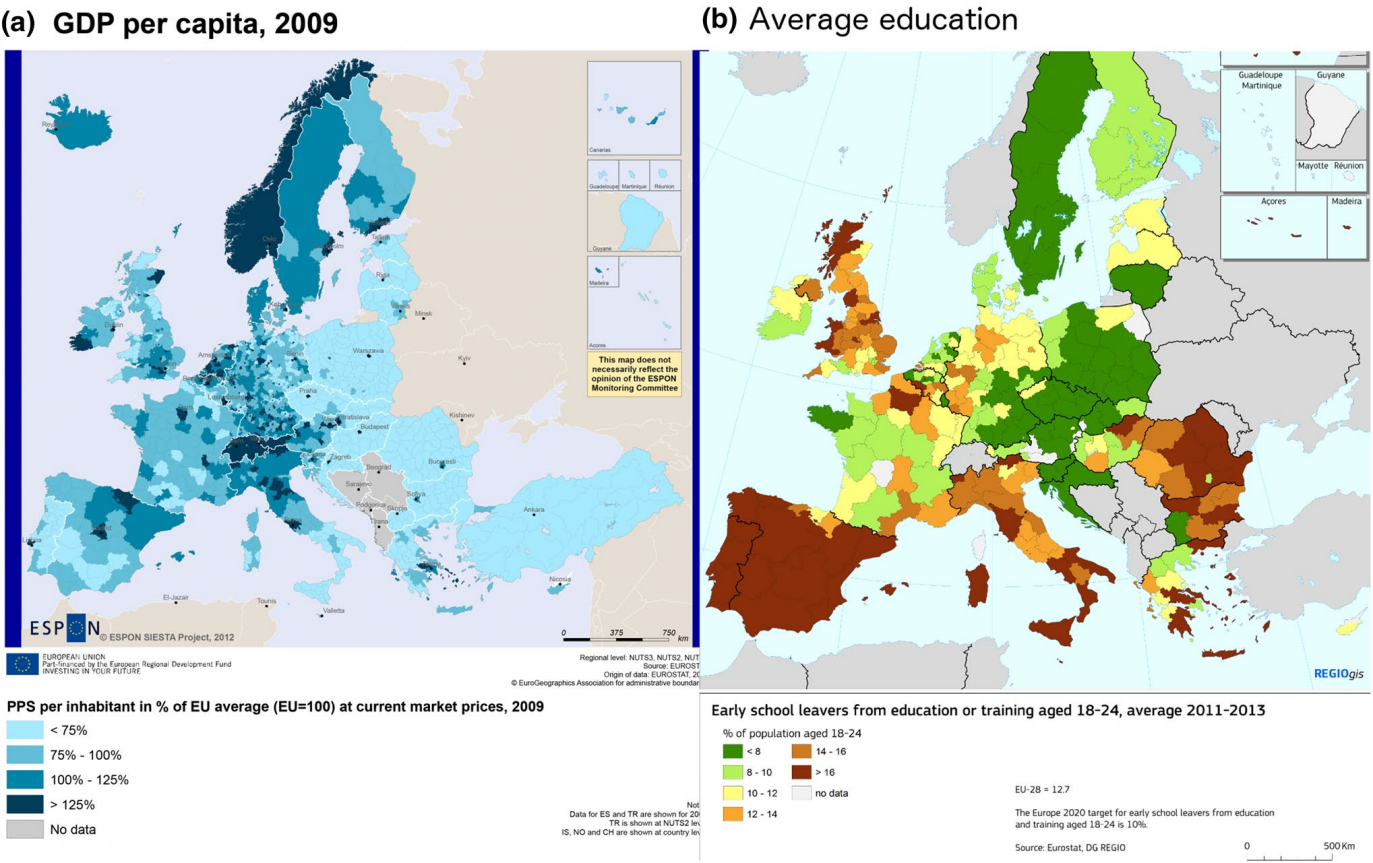
Maps of measures of educational attainment correlate with GDP, both within and across countries in Europe. There are large differences between North and South Europe, and this is plausibly associated with genetic ancestry. This may confound inference by generating genetic associations with education that are not biologically causal but are instead driven by access to education. Data source: Eurostat http://ec.europa.eu

It is likely that a combination of the above is true. Non-causal pathways are certainly plausible (Fig. 5): average education levels and GDP per capita are correlated within countries and between countries in Europe (Mankiw et al. 1992). GDP is in turn correlated in Northern Europe and the UK with high Germanic and Scandinavian ancestry, such as England, Germany, Denmark, Netherlands, Belgium and Luxembourg.

The difference in selection signal for height between UK Biobank and GIANT (Figure 1 of Berg et al. 2018) cannot be explained by PC correction, as the difference in effect exists even in uncorrected UK Biobank estimates. Stratification may play a role, but it is more complex than a simple correction issue.

We saw in the stroke example that having access to multiple populations is transformative for how SNP effects can be interpreted. This genetic and cultural diversity is helpful in the study of all phenotypes, though we also need new methodology to further exploit the rich information available from these datasets. It may be the case that some phenotypes—including educational attainment and psychiatric disorders—are associated with traits that are actually creating the observed population structure. The arrow of causality then becomes unclear, and we may need dynamical models for historical data to complete the picture.

### Epidemiology has much to gain from recognizing population structure

Stratified, admixed and otherwise heterogenous populations are an opportunity to test and validate the statistical models built on inferred genetic contributions to traits.

We also need to revise the data-sharing practices that we use. There are at least three different ways to run GWAS, which might all inform an understanding of how a SNP is associated with a phenotype:

1. *Unadjusted estimates:* These are likely to individually be overestimates of the causal effect and contain false positives.
2. *PCA- or LMM-adjusted estimates:* In many cases, these can individually be underestimates of the causal effect.
3. *Externally adjusted estimates:* Many of the problems discussed would disappear by standardizing the correction: for example, if small studies standardize against genetic variation in the UK Biobank then under-correction will be reduced. These PCs can be included alongside the standard PCs which would still be required to correct for batch effects, residual family structure, etc.

None of these approaches is “correct” for estimating the “true” causal effect sizes for individual SNPs, but having varied estimates from varied populations allows for triangulation (Lawlor et al. 2016) and hence brings us towards a better understanding of the underlying relationship between genotype and phenotype. The third approach may be particularly important for the standardization of varied sizes of study included in meta-analysis.

This article has discussed the part that population structure may play in the future of genetic epidemiology. Observational relationships between genetic variants and phenotypes are validated through external measurements—structured populations and structured environments. Examining robustness of association signals in different populations is just one form of triangulation, and external validation in non-human models, in vitro experiments and clinical trials all will continue to play a key role.

### Box 1: Structure, stratification, ancestry, demography, kinship?

Individuals are not randomly sampled from a large homogeneous population. *Population structure* is the existence of correlated variation in allele frequencies between (sub)populations, meaning that SNPs on different chromosomes are predictive of each other. This can lead to *phenotypic population stratification* or “allele frequency differences < associated with phenotype > due to systematic ancestry differences” (Price et al. 2006). *Ancestry* refers to the proportion of the genome that individuals received from historical abstracted populations, which change over time and are related through their *demography*. Populations and their history are a modeling construct that makes sense of the family tree (or pedigree) relating all individuals that left descendants in the sampled individuals. This is often measured through a *kinship* matrix or genetic similarity between all individuals in a dataset. Principal component analysis (PC analysis) is a dimension-reducing method to focus on ancestry by measuring the largest-scale variations in kinship, but the full kinship matrix also measures recent relatedness.

Correctly accounting for structure is important. If phenotypic stratification is insufficiently accounted for, then variants associated with population structure become associated with stratified phenotypes: for example, LCT variation is correlated not just with Lactase persistence but also with height (Campbell et al. 2005).

## Methods

### Model for simulating genotypes in varying environments

This simulation is designed to describe prediction quality in a range of situations where admixture has led to a single, relatively homogeneous population from two source populations, which are different in SNP frequency for some reason (either selection or drift) that is not explicitly modeled. It treats environment as separate from ancestry, so that a “test population” can be constructed in a different environment to the training population.

We simulated a sample of N individuals at L SNPs. To construct a model including correlations between PCs and traits, we allow individuals to be admixed between two populations (*j* = 1, 2). We then increase the frequency of the SNPs that are associated with the trait within Population 1. Finally, we add an environmental confounder associated with the admixture proportion from Population 1. This leads to a situation in which genes are associated with phenotype via two pathways, a “causal” genetic pathway and a “non-causal” environmental confounded pathway.

Under these conditions, prediction accuracy is reduced by PC correction (because PCs are associated with the genetics of the trait). However, the raw predictor is fitting the environmental component as well as the genetic. The environmental effect is removed by correcting for PCs. This lowers the prediction accuracy.

Specifically, the admixture fraction for individual $i$ from Population 1 is:

$$a_{i} \sim TruncNorm\left( {a_{0} ,\sigma_{\text{a}}^{2} } \right),$$

where $\sigma_{\text{a}}^{2} = a_{0} \left( {1 - a_{0} } \right)/L_{\text{eff}}$ represents the variance expected were the admixture fraction to be sampled under a binomial with $L_{\text{eff}}$. This simulates recent (small $L_{\text{eff}}$) or ancient (large $L_{\text{eff}}$) admixture. $a_{i}$ are truncated to lie within (0,1), allowing admixture proportions of exactly 0 or 1 to be simulated.

We then simulate ancestral the allele frequency for SNP $l$ as $p_{\text{l}} \sim Uniform(0.05,0.5)$. Population SNP frequencies are $p_{\text{lm}} = TruncNorm\left( {p_{\text{l}} \left( {1 + c_{i} s} \right),p_{\text{l}} \left( {1 - p_{\text{l}} } \right)\sigma_{\text{p}}^{2} } \right)$, where $\sigma_{\text{p}}^{2}$ describes genetic drift from the ancestral frequency, $s$ approximates a “selection” or extreme drift effect for SNPs associated with the trait, and $c_{\text{l}} = 1$ for SNPs that are causal for the trait and 0 otherwise. Truncation allows frequencies of exactly 0 or 1 to be simulated.

The effect sizes are $\beta_{\text{l}} \sim Uniform\left( {0,c_{\text{l}} \beta_{0} p_{\text{l}} \left( {1 - p_{\text{l}} } \right)} \right)$. The SNP data $X_{\text{il}}$ are then sampled $X_{\text{il}} \sim bern\left( {a_{i} p_{{{\text{l}}1}} + \left( {1 - a_{i} } \right)p_{{{\text{l}}2}} } \right)$. The genetic contribution to the phenotype is $Y_{i}^{\text{G}} = \sum\nolimits_{l = 1}^{L} {X_{il} \beta_{l} }$ and standardize $Y_{i}^{\text{G}}$ to have mean 0 and variance 1. We then generate an environmental exposure $E_{i} = (a_{i} - a_{0} )/\sigma_{\text{a}}$ with expected variance 1.

Finally, we construct a final phenotype $Y_{i} = hY_{i}^{\text{G}} + \left( {1 - h} \right)eE_{i} + \left( {1 - h} \right)\left( {1 - e} \right) \in_{i}$, where $\in_{i}\; \sim Norm\left( {0,1} \right)$, $h$ describes the “heritability” of the trait and $e$ describes the “environmental contribution”. $Y_{i}$ is therefore a mixture of components with mean 0 and variance 1 by construction.

To generate the plots, we simulate data under this model, and then examine new phenotypes in new data generated under the same model with estimated $\widehat{\beta }_{\text{l}}$ from the function “mixed.solve” in the R package rrBLUP. The top 20 PCs are calculated using the function “irlba” in the package irlba.

Simulations use $h = 0.5,\;e = 0.5$, $N = 2000$, $L = 4000$, $L_{\text{eff}} = 20$, $a_{0} = 0.4,\;\beta_{0} = 1$ and $s = 0.2$ by default. Sensitivity analysis shows that no conclusions are dependent on the details of these choices (not shown).

### Modeling the ALSPAC data

We created a combined dataset of PoBI and ALSPAC mothers, which is described fully in Haworth et al. (2018). Briefly, these were jointly phased, and used the imputed genotypes of ALSPAC participants at a set of 508,223 SNPs chosen by Leslie et al. (2015) for the PoBI dataset. Chromosome painting (Lawson et al. 2012) was used to find the haplotypes that each individual shared with each of the 35 labeled populations from the PoBI dataset (Fig. 2). The PoBI data forms a reference dataset for which a mixture model is fit for ALSPAC mothers. The map in Fig. 2a shows what the genetic score for that region would be, should the observed education in ALSPAC mothers be generated by mixing their regional education values by the inferred admixture weights.

A singular value decomposition (SVD) was then applied to the *N* = 2039 by *K* = 35 matrix of the results of chromosome painting for the PoBI participants (after centering and standardizing variance). Each of the *M* = 7739 mothers for whom genetic and education data were available were mapped into the SVD. Variance explained (Fig. 2b) uses a linear model predicting education either from chromosome painting or raw genotype PCs.

The heatmap (Fig. 2c) is a 2D smoothing of education values using chromosome painting PC3 and PC8, using “predict.gam” from the R package “mgcv” (restricted to where there is an observation within a distance 0.03).
